# Microbiome Profiling Reveals Gut Dysbiosis in the Metabotropic Glutamate Receptor 5 Knockout Mouse Model of Schizophrenia

**DOI:** 10.3389/fcell.2020.582320

**Published:** 2020-10-29

**Authors:** Carolina Gubert, Geraldine Kong, Volkan Uzungil, Ariel M. Zeleznikow-Johnston, Emma L. Burrows, Thibault Renoir, Anthony J. Hannan

**Affiliations:** ^1^Florey Institute of Neuroscience and Mental Health, The University of Melbourne, Parkville, VIC, Australia; ^2^Department of Anatomy and Neuroscience, The University of Melbourne, Parkville, VIC, Australia

**Keywords:** gut dysbiosis, microbiota, microbiome, psychiatric disorders, schizophrenia, mGlu5 knockout mice

## Abstract

Schizophrenia (SZ) is a psychiatric disorder that constitutes one of the top 10 global causes of disability. More recently, a potential pathogenic role for the gut microbial community (microbiota) has been highlighted, with numerous studies describing dysregulated microbial profiles in SZ patients when compared to healthy controls. However, no animal model of SZ has previously recapitulated the gut dysbiosis observed clinically. Since the metabotropic glutamate receptor 5 (mGlu5) knockout mice provide a preclinical model of SZ with strong face and predictive validity, in the present study we performed gut microbiome profiling of mGlu5 knockout (KO) and wild-type (WT) mice by 16S rRNA sequencing of bacterial genomic DNA from fecal samples, analyzing bacterial diversity and taxonomic composition, as well as gastrointestinal parameters as indicators of gut function. We found a significant genotype difference in microbial beta diversity. Analysis of composition of microbiomes (ANCOM) models were performed to evaluate microbiota compositions, which identified a decreased relative abundance of the *Erysipelotrichaceae* family and *Allobaculum* genus in this mouse model of SZ. We also identified a signature of bacteria discriminating between the genotypes (KO and WT), consisting of the Erysipelotrichales, Bacteroidales, and Clostridiales orders and macroscopic gut differences. We thus uncovered global differential community composition in the gut microbiota profile between mGlu5 KO and WT mice, outlining the first evidence for gut dysbiosis in a genetic animal model of SZ. Our findings suggest that this widely used preclinical model of SZ also has substantial utility for investigations of gut dysbiosis and associated signaling via the microbiota–gut–brain axis, as potential modulators of SZ pathogenesis. Our discovery opens up new avenues to explore gut dysbiosis and its proposed links to brain dysfunction in SZ, as well as novel therapeutic approaches to this devastating disorder.

## Introduction

Schizophrenia (SZ) is a devastating psychiatric disorder characterized by positive (e.g., hallucinations) and negative (e.g., reduced motivation) symptoms, and cognitive deficits, which existing pharmacological treatments generally fail to comprehensively treat (Leung et al., 2019). While it affects both men and women, there are sex differences in SZ, with men showing an earlier age at onset together with higher propensity to negative symptoms, co-morbid substance abuse and lower social functioning (reviewed in Li et al., 2016). Importantly, chronic gastrointestinal (GI) tract issues exist as comorbid symptoms of SZ, including gut inflammation (Severance et al., 2015; McCutcheon et al., 2020).

More recently a potential role for the microbial community that resides in the gut (called gut microbiota) in SZ pathogenesis has been highlighted, with multiple groups describing dysregulated microbial profiles in SZ patients when compared to healthy controls (Castro-Nallar et al., 2015; Schwarz et al., 2018; Shen et al., 2018; Xu et al., 2019; Szeligowski et al., 2020). Disruption of microbiota can lead to gut microbial imbalance, called gut dysbiosis, and this condition has been shown to occur in various brain disorders, including SZ (Cryan and Dinan, 2012). Gut dysbiosis not only means a differential gut microbial population constituting a pathogenic profile but is also associated with impairment in gut integrity, functionality, intestinal permeability, as well as gut inflammation, and thus an aberrant gut environment. This profile generates a milieu of signaling molecules that ultimately can communicate with the brain through neural communication (via the vagus nerve), endocrine signaling, the immune system and microbial metabolites, thus modulating brain function, and, most remarkably, cognition (reviewed in Gubert et al. (2020)).

Due to discrepancies between clinical studies regarding specific taxa abundance and enrichment, a unique gut microbiome profile for SZ patients is still not determined (Macedo e Cordeiro et al., 2020). However, several clinical studies using different approaches in order to determine the SZ microbiota profiling or signature by determining the population diversity and taxonomic composition or bacterial abundance have been performed, revealing a potential new etiological aspect of SZ associated with an overall gut dysbiosis (reviewed by Rodrigues-Amorim et al., 2018). In fact, recent studies suggest that the disruption of the microbiota–gut–brain axis may promote the development of SZ and additionally, a few bacterial taxa including *Veillonellaceae* and *Lachnospiraceae* may associate with SZ severity (Zheng et al., 2019). Further evidence that SZ microbiota may be disease-causing is provided by a metagenome-wide association study showing differences in short-chain fatty acids (SCFAs), and neurotransmitter metabolism, synthesis and degradation in patient microbiota (Zhu et al., 2020). Given the emerging evidence supporting a role for gut dysbiosis in SZ pathogenesis, there is great potential to exploit this as an intervention target, as microbiota can be modulated via a range of therapeutic approaches.

Mice are frequently used to probe mechanistic questions relating to complex brain disorders, including in gut microbiota research (Nguyen et al., 2015). In several studies, fecal matter transplant of gut microbiota from SZ patients has been reported to result in behavioral changes in mice reminiscent of SZ symptoms (Zheng et al., 2019; Zhu et al., 2019, 2020). Interestingly, germ-free mice receiving SZ microbiome fecal transplants demonstrated lower glutamate and higher glutamine and GABA while SZ-relevant behavior in mice (Zheng et al., 2019). However, despite clinical evidence, no animal model of SZ has previously modeled gastrointestinal dysfunction or dysbiosis, although gut dysbiosis has been characterized in animal models of other psychiatric disorders, such as depression (Cheung et al., 2019; Sun et al., 2019).

In the present study, we aimed to profile the gut microbiome of a well characterized mouse model of relevance to SZ, metabotropic glutamate receptor 5 (mGlu5) knockout mice and their wild-type (WT) controls (Gray et al., 2009; Burrows et al., 2015; Zeleznikow-Johnston et al., 2018). Mouse models of schizophrenia are scrutinized against construct, face and predictive validity. While construct validity, or how well an animal model recapitulates the etiology, is difficult to achieve given the complex genetic and environmental drivers of SZ, mGlu5 KO mice model glutamatergic disruption hypothesized to contribute to the disorder (Devon et al., 2001; Gupta et al., 2005) and modulators of the receptor have been of interest in the context of treatment (Balu et al., 2016; Maksymetz et al., 2017; Stansley and Conn, 2018; Uno and Coyle, 2019). The mGlu5 KO mouse model exhibits abnormal brain maturation (Hannan et al., 2001) and a behavioral phenotype that mimics the positive and cognitive symptoms of SZ. Specifically this includes impairments in pre-pulse inhibition (a translational measure of sensorimotor gating in SZ patients and mice), hyperlocomotion (proxy for psychomotor agitation in psychosis) and short-term and long-term spatial memory deficits (Brody and Geyer, 2004; Brody et al., 2004; Gray et al., 2009; Xu et al., 2009; Bird et al., 2014; Burrows et al., 2015; Zeleznikow-Johnston et al., 2018; Lim et al., 2019; Aguilar et al., 2020). Predictive validity refers to response to a clinically effective treatment and beneficial effects on both positive and cognitive symptoms have been shown in mGlu5 mice following treatment with the antipsychotic drug clozapine (Gray et al., 2009).

To date, no study has scrutinized gut health in mGlu5 KO mice or any other mouse model of relevance to SZ, and we hypothesized that differences in the gut microbiome would exist between the two genotypes. To characterize the general gut health, we have performed microbiome profiling using 16S rRNA sequencing, analyzing bacterial diversity and taxonomic composition, as well as gastrointestinal parameters as indicators of gut function. The overall goal of this study was to establish whether this SZ mouse model could be utilized as an appropriate tool for preclinical study of gut dysbiosis.

## Materials and Methods

### Animal Husbandry

Wild-type (WT) and mGlu5 KO male mice (Grm5tm1Rod; Lu et al., 1997) were generated from heterozygous breeding pairs that had been maintained past generation F10 on a C57Bl/6 background. Genotypes were determined by PCR, from a tail biopsy. The mice were co-housed according to genotype after weaning at 4 weeks of age, in order to avoid potential sharing of microbiota due to the coprophagic nature of mice, unless indicated. The housing condition consisted of open-top standard mouse cages (34 × 16 × 16 cm^3^; 2–4 mice/box) with basic bedding and nesting materials. Cage changes were performed weekly.

For 16S rRNA sequencing and body weight assessment the same cohort of mice was used, with a total of 6 mGlu5 KO and 6 WT mice, both genotypes split between two cages. All mice had *ad libitum* access to food and water and were housed in a controlled room of 22°C of temperature and 45% of humidity on a 12:12 h light/dark cycle. All procedures were approved by The Florey Institute of Neuroscience and Mental Health Animal Ethics Committee and were performed in accordance with the relevant guidelines and regulations of the National Health and Medical Research Council Code of Practice for the Use of Animals for Scientific Purposes.

### Food and Water Intake

Two extra cohorts were generated and used to assess food and water intake. While for the water intake measures, those mice were still separated by genotype, for food intake measures the respective mice were housed in mixed genotype groups (2–4 mice per box). Cumulative food and water intake were assessed, both over 4 weeks in grouped-housed mice, with the intake normalized to body weight to account for variability. These results are represented as mg of food per g of body weight and as g of water per g of body weight, respectively, to cumulative food and water intake.

### Gastrointestinal Measurements

A separate cohort of 7 WT and 6 mGlu5 KO mice at 26 weeks of age was used in order to determine gastrointestinal parameters.

#### Fecal Output and Fecal Water Content

Fecal output and fecal water content were assessed by single caging the animals for 1 h and counting the number of fecal pellets expelled during this period. All the fecal pellets were collected, and the total weight was measured before being dried at 95 °C for > 3 h. The percentage of difference between the initial total feces weight and the dry weight is taken as fecal water content.

#### Gastrointestinal Transit Time

Gastrointestinal transit time was determined using the non-absorbable red dye Carmine red (Sigma-Aldrich), prepared as a 6% (w/v) solution in 0.5% methylcellulose (Sigma-Aldrich) filtered and autoclaved prior to use. Non-fasted mice were gavaged with 150 μL of the carmine solution and housed individually. The time taken from gavage to the first appearance of carmine in the feces was recorded as the total transit time.

#### FITC-Dextran Intestinal Permeability

Intestinal epithelial barrier permeability was assessed by 4 kDa fluorescein isothiocyanate (FITC)−dextran (Sigma-Aldrich). Mice were fasted for 4 h prior to oral gavage with 150 μL FITC-dextran (dissolved in PBS to a concentration of 100 mg/mL). Blood was collected 4 h post administration, and immediately transferred to an EDTA collection tube and further centrifugated at 1,000 × g for 10 min. Plasma was collected and fluorescence was quantified at an excitation wavelength of 485 nm and 528 nm emission wavelength (PHERAstar *FSX*, Millipore). Serial dilutions of FITC-dextran in PBS were used to calculate a standard curve.

#### Macroscopic Assessment

Mice were euthanized by cervical dislocation, and their intestines were excised. The intestine was placed in a non-absorbent surface and the length of cecum and colon was measured using a ruler. The weight of cecum with their contents was also evaluated. All of these parameters were normalized to the body weight (g) of the animals, unless indicated.

### Fecal Sample Collection and DNA Extraction for 16S rRNA Sequencing

At 12 weeks of age, fecal samples were collected. Mice were individually placed in clean cages for up to 10 min. Fresh pellets were collected and immediately frozen in dry ice and stored at −80°C until further processing.

The fecal genomic DNA was extracted using the PowerSoil HTP kit (Qiagen). The number of sequences per sample was determined as the standard quality control metric (Supplementary Table 1), which showed a mean of approximately 75,500 sequences across all the samples. The extracted genomic DNA and amplified using universal prokaryotic 515F and 806R primers targeting the V4 hypervariable region of the 16S small subunit rRNA gene (Caporaso et al., 2011). Amplicon 16S rRNA gene sequences were generated using paired-end 150 bp sequencing on the Illumina MiSeq platform.

### 16S rRNA Sequencing, Bioinformatics, and Statistical Analysis

Illumina MiSeq raw FASTQ data were processed using Qiita for quality control, demultiplexing sequences, trimming, and resolving exact sequence variants (ESVs) or Amplicon Sequence Variants (ASVs) with Deblur (Amir et al., 2017). The representative sequences were mapped onto Silva_132 99% to obtain the taxonomic identity of ASVs (Quast et al., 2012). Downstream analyses were computed using R software version 3.5.2.

We first measured the alpha-diversity, which summarizes the diversity of microbial structure within a sample. Reads were rarefied to 1100 reads (Supplementary Figure 1), which is the global minimum of the number of reads in the sequencing data, to compute several alpha-diversity metrics, including species richness (Observed), Shannon, Inverse Simpson and Fisher metrics, using the “Phyloseq” R package (McMurdie and Holmes, 2013). Species richness (Observed) is the number of operational taxonomic units (OTU) observed in a given sample. Shannon, Inverse Simpson and Fisher diversity metrics are a composite measure of richness (number of OTUs present) and evenness (relative abundance of OTUs). Kruskal–Wallis test was used to compare the species richness and alpha-diversity measurements between the genotypes.

To estimate beta-diversity, which summarizes the diversity between samples, the counts were normalized to their relative abundance by dividing raw counts from a particular sample by the total number of reads in each sample. We applied a relative abundance cut-off of 0.01% on the data (including only the relative abundance > 0.01% of the data detected), resulting in a total of 202 ASVs for the subsequent analysis. Two measures of beta-diversity, Bray-Curtis and unweighted UniFrac distances (Lozupone and Knight, 2005), were calculated and used in principal coordinates analysis (PCoA). The unweighted UniFrac distance accounts for the phylogenetic relationship between the OTUs, whereas the Bray-Curtis distance accounts for the abundance of the OTUs. To determine whether the visually observed differences were statistically significant, Adonis (Permutation multivariate ANOVA—PERMANOVA) from the “vegan” R package was performed with 999 permutations (Anderson, 2001; Dixon, 2003). The *R*^2^ value reported by Adonis PERMANOVA indicates the amount of variance, on a scale of 0 to 1, in the data which can be explained by the factors tested.

Before testing for differential abundance in the various taxonomic levels, centered log-ratio (CLR) transformation was applied to account for compositionality in microbiome data. Analysis of composition of microbiomes (ANCOM) was used to identify differential relative abundance of each bacterial family and genus between genotypes (Mandal et al., 2015). ANCOM accounts for the compositional nature of the taxa relative abundances and reside on the analysis of difference in pairwise log-ratio while controlling for false discoveries. We applied ANCOM with FDR correction of 0.05. A high “w score” generated by this test indicates the greater likelihood that the null hypothesis can be rejected, indicating the number of times a parameter is significantly different between groups.

Moreover, sparse PLS discriminant analysis (sPLS-DA) from the “mixOmics” package in R was used to identify a signature of discriminative ASVs associated with genotype (Lê Cao et al., 2016; Rohart et al., 2017). The performance of the model is evaluated using the leave-one-out cross validation method.

### Co-occurrence Network Analysis

Given that microbiome data is compositional and transformation from absolute to relative abundances introduces spurious correlations, Sparse Correlations for Compositional data (SparCC) was employed to calculate correlations between ASVs to build the co-occurrence network (Friedman and Alm, 2012). SparCC and calculation of two-sided pseudo p values were performed based on bootstrapping of 100 repetitions. Correlations with p-values of < 0.05 and magnitude of = 0.6 (indicating strong co-abundance relationships) or =−0.6 (indicating strong exclusion relationships) were used to construct a network using qgraph (Epskamp et al., 2012). Clusters within the network were determined using the walktrap algorithm with 4 steps implemented in igraph (Csardi, 2006). The network was then visualized and plotted in Cytoscape 3.8 using a customized layout (Shannon, 2003).

### General Statistical Analysis

In addition to the gut microbiome analyses described above, to verify the effects of genotype on the body weight, food intake, and water intake, unpaired *t*-tests were performed. These analyses and respective graphs were constructed using Graph^®^ Pad Prism 8 software. In all cases, significance level was set to *p* < 0.05.

## Results

### Gut Dysbiosis in mGlu5 KO Mice

To characterize the gut microbiome, the genomic DNA from feces collected at 12 weeks of age was extracted for 16S amplicon sequencing. Estimates of alpha and beta diversity, both indicative of differences in microbial communities, were obtained using a bioinformatic approach to cluster genetic data from mGlu5 KO and WT mice samples.

**FIGURE 1:**
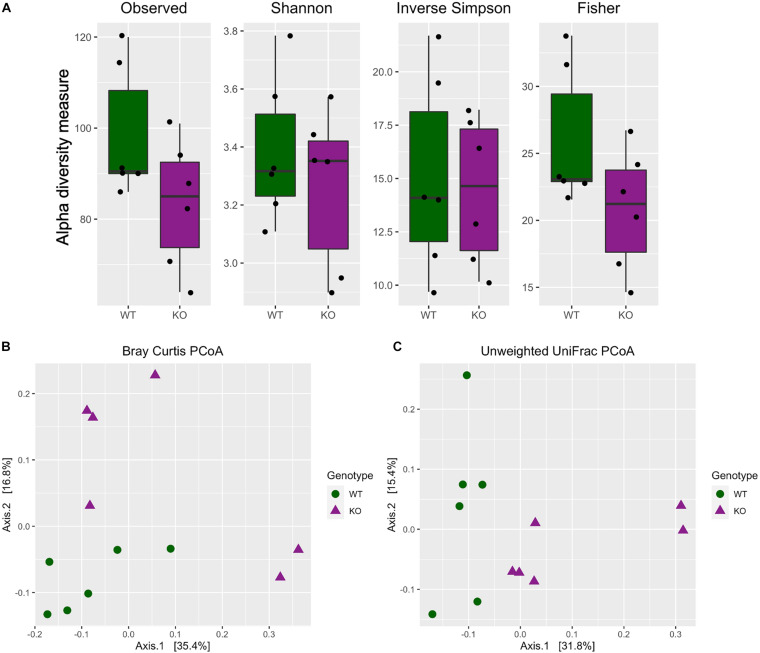
Differential microbiota diversity profiling in mGlu5 KO mice. **(A)** Alpha diversity metrics including Observed, Shannon, Inverse Simpson (InvSimpson), and Fisher indices. There was no difference in all four indices between WT and mGlu5 KO mice (Kruskal–Wallis test). Beta diversity principal coordinate analysis of WT and mGlu5 KO data using **(B)** Bray-Curtis distance and **(C)** unweighted UniFrac distances. The samples were clustered according to the genotype, and there were significant differences between the two genotypes in both Bray-Curtis and unweighted UniFrac distances. Permutation multivariate ANOVA is (*n* = 6) for both WT and mGlu5 KO mice groups).

The alpha diversity indices (Observed, Shannon, Inverse Simpson, and Fisher) were calculated and plotted to visualize the difference between the two genotypes. Further statistical testing using Kruskal–Wallis method revealed no significance in any of the alpha diversity indices measured [Figure 1A, Kruskal Wallis test, Observed (*H* = 2.08, df = 1, *p* = 0.1488); Shannon (*H* = 0.1, df = 1, *p* = 0.7488); Inverse Simpson (*H* = 0.1, df = 1, *p* = 0.7488); Fisher (*H* = 2.08, df = 1, *p* = 0.1488)].

For beta diversity, both Bray-Curtis and unweighted UniFrac distance were calculated and the samples were ordinated based on those distances. When the entire population was examined together on a PCoA plot, using both the Bray-Curtis and unweighted UniFrac distance, samples tended to cluster according to the genotype. For Bray-Curtis PCoA, the stratification of samples according to groups was mostly on the second component (Axis 2), accounting for approximately 17% of variation in the data (Figure 1B; Bray Curtis dissimilarity distance _*Permanova*_*R*^2^ = 0.19, *p* = 0.021). Whereas for unweighted UniFrac PCoA, the stratification of samples according to groups was mostly on the first component (Axis 1), accounting for approximately 32% of variation in the data (Figure 1C; unweighted UniFrac distance _*Permanova*_*R*^2^ = 0.23, *p* = 0.003).

**FIGURE 2:**
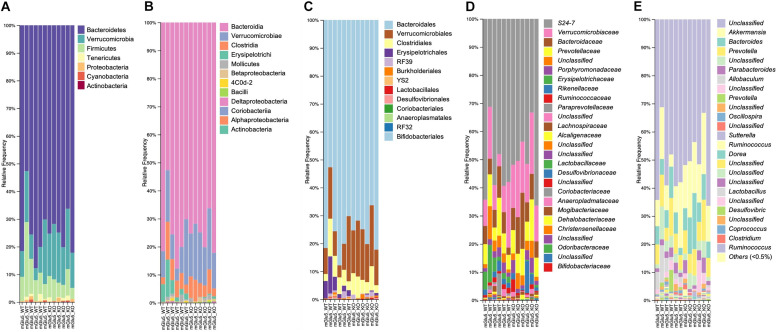
Taxonomic composition distribution histograms of each mGlu5 KO and WT sample. At **(A)** Phylum level, **(B)** class level, **(C)** order level, **(D)** family level, and **(E)** genus level. *n* = 6 for both WT and mGlu5 KO mice groups.

Across all mice, the most abundant Phyla was Bacteroidetes (74.9%), followed by Verrucomicrobia (14.6%), and then the Firmicutes (9.0%), which together made up approximately 98.5% of total abundance (Figure 2A). At Class level, the most abundant were Bacteroidia (74.8%), Verrucomicrobiae (14.5%), Clostridia (5.5%) and Erysipelotrichi (3.5%), which together are 98.3% of total abundance (Figure 2B). In addition, the most abundant Order were Bacteriodales (74.8%), Verrucomicrobiales (14.5%), Clostridiales (5.5%) followed by Erysipelotrichales (2.6%) (Figure 2C). A total of 27 bacterial families were detected in genomic data, and 23 remained after passing through a relative abundance cut-off of 0.01% (Figure 2D). The most abundant bacterial Family in its turn was *S24.7* (from Bacteriodales order), comprising 51.5% of the bacterial population, followed by *Verrucomicrobiaceae* (14.6%), *Prevotellaceae* (8.5%), *Bacteroidaceae* (8.5%), and *Erysipelotrichaceae* (2.6%). Finally, a total of 39 genus were detected and 32 of these passed the cut off relative abundance of 0.01% (Figure 2E). The most abundant genus was *Unclassified* (from *S24.7* family) with 51.4% of the bacterial population, followed by *Akkermansia* (14.5%), *Bacteroides* (8.5%), and *Prevotella* (8.4%).

**FIGURE 3:**
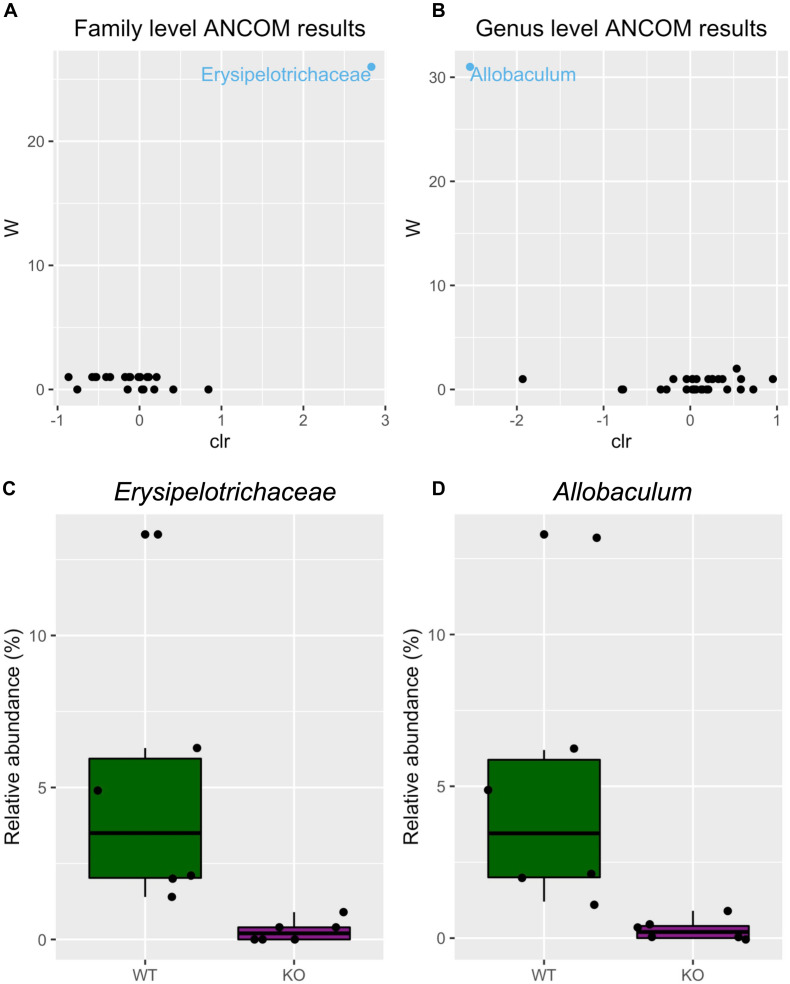
Differential microbiota abundance in mGlu5 KO mice. Volcano plot for the analysis of composition of microbiomes (ANCOM) test for **(A)** family and **(B)** genus level. Only significant bacterial taxa are labeled and colored in blue. Significant taxa are showing high w-stats. Boxplots comparing the relative abundance of the family and genus whose abundance was found to differ significantly between mGlu5 KO and WT **(C)** *Erysipelotrichaceae* and **(D)** *Allobaculum*. The plots show mean ± SEM (*n* = 6 for both WT and mGlu5 KO mice groups).

Next, we tested differential abundance of bacteria between mGlu5 KO and WT, using analysis of composition of microbiomes (ANCOM), which identified the family *Erysipelotrichaceae* (Figure 3A) and the genus *Allobaculum* (Figure 3B) as having different levels between genotypes. The relative abundance of these bacteria was identified as being decreased in both family (Figure 3C) and genus (Figure 3D) in mGlu5 KO mice when compared to WT littermate controls.

### Signature of Bacteria Discriminating Both Genotypes (KO and WT)

**FIGURE 4:**
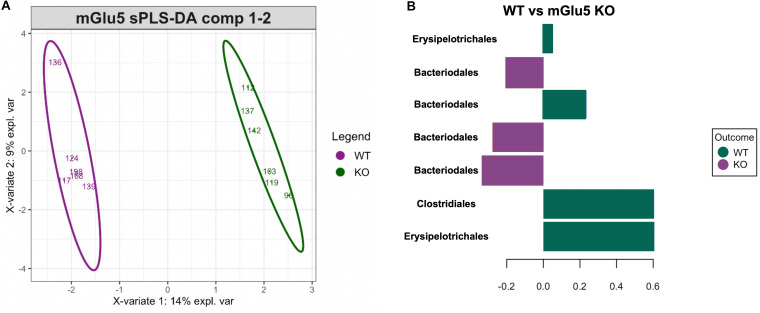
Identification of a bacterial signature discriminating the genotypes with sPLS-DA. **(A)** Sample plots with 0.95 confidence ellipse plots for WT and mGlu5 KO show a strong discrimination between genotypes. The top 7 resulting bacterial signature contributing to component 1 is displayed in **(B)**. The length of the bars indicates the importance of each amplicon sequence variants (ASVs) (at the Order taxonomic rank) in the signature (from bottom to top: decreasing importance) with color indicating the phenotype group with maximum median abundance (n = 6 for both WT and mGlu5 KO mice groups, classification error rate = 0.11).

In order to detect specific ASVs which could contribute to the stratification of samples according to their genotype (as seen above on the PCoA), sPLS-DA was performed (Figure 4A). sPLS-DA is a multivariate method performed on the clr-transformed microbiome data to identify microbial drivers discriminating particular phenotype groups. The method identified a signature of bacteria discriminating WT and mGlu5 KO mice, detecting 7 ASVs whose respective contribution is shown on the loading plot (classification error rate = 0.11, Figure 4B). For WT mice, this signature consisted of the Erysipelotrichales, Bacteroidales and Clostridiales order. For mGlu5 KO, this signature consisted of bacteria from the Bacteroidales order.

### SparCC-Derived Co-abundance Network Analysis

To obtain a measure of association between ASVs while incorporating their abundance, we used SparCC correlation coefficients that are robust for analyzing compositional microbiome data. A total of 20,301 associations were found, and 78 associations with a p value of less than 0.05 were observed in the network: 46 (59%) of those were positive (*r* = −0.6) and 32 (41%) were negative (*r* =−0.6).

**FIGURE 5:**
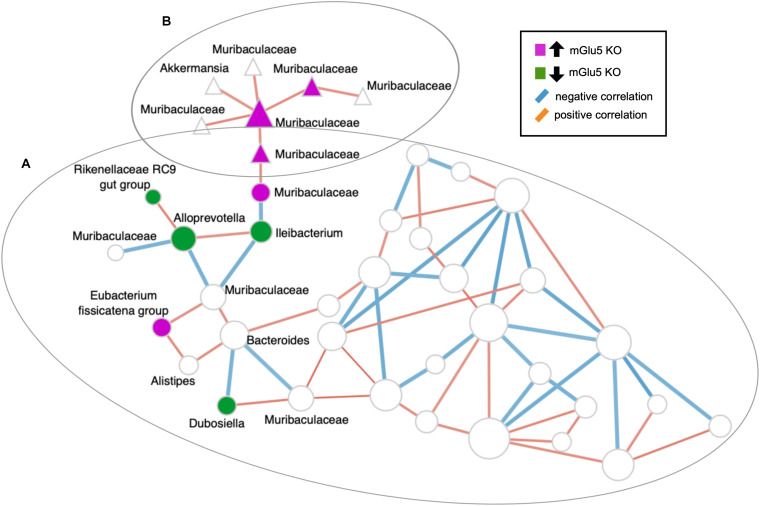
SparCC-derived co-abundance network analysis. Nodes belonging to the same clusters calculated using the walktrap algorithm are depicted by their shapes (all nodes with circles belong to cluster **(A)**, all nodes with triangles belong to cluster **(B)**). Nodes that were detected by sPLS-DA and their first neighbors were labeled. Nodes colored in magenta are amplicon sequence variants (ASVs) detected by sPLS-DA to be increased in KO mice, while the nodes colored in dark green are ASVs detected by sPLS-DA to be decreased in KO mice. ASVs are shown at the Genus level, in the case where Genus is not assigned, the next highest taxonomy level is shown. The edges (lines connecting two circles) are colored according to their correlation: blue for negative correlation, red for positive correlation. Isolated nodes are omitted from the network.

The top 10 keystone taxa (taxa with the most interactions with other taxa) detected in this network are ASV_377, ASV_361, ASV_252, ASV_107, ASV_181, ASV_223, ASV_159, ASV_105, ASV_287 and ASV_99 (none of which are detected by the sPLS-DA). Articulation points are defined as nodes in which when removed with its associated edges, makes the graph disconnected. Some of the articulation points in the network include the ASVs also detected by sPLS-DA: including ASV_557 (genus *Ileibacterium*), ASV_138 (family *Muribaculaceae*), ASV_134 (family *Muribaculaceae)*, and ASV_275 (family *Muribaculaceae)* (Figure 5).

### Reduced Body Weight in mGlu5 KO Mice Despite No Changes in Food and Water Intake

**FIGURE 6:**
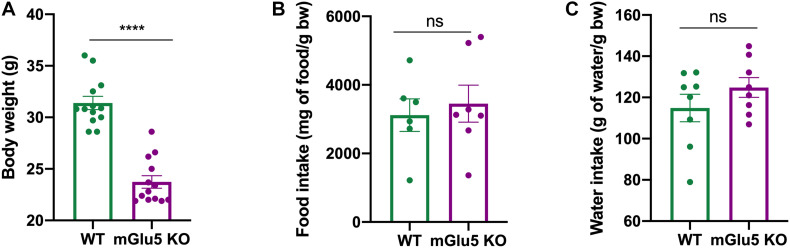
Reduced body weight in mGlu5 KO mice despite no changes in food and water intake. **(A)** Decreased body weight in mGlu5 KO mice compared to WT at 12 weeks of age, unpaired *t*-test. **(B)** Cumulative food intake (represented as mg of food per g of body weight) and **(C)** cumulative water intake (represented as g of water per g of body weight), both over 4 weeks between mGlu5 KO mice and WT controls, unpaired *t*-test. The plots show mean ± SEM (*n* = 6–8 for both WT and mGlu5 KO mice groups). ***p < 0.001.

mGlu5 KO mice presented a reduction in body weight when compared to WT controls (Figure 6A; *t* = 5.72, df = 10, *p* = 0.0002). No differences were found in food (Figure 6B; *t* = 0.04, df = 11, *p* = 0.65) or water (Figure 6C; *t* = 1.21, df = 14, *p* = 0.2) intake between mGlu5 KO mice and WT controls.

### Gastrointestinal Parameters

**FIGURE 7:**
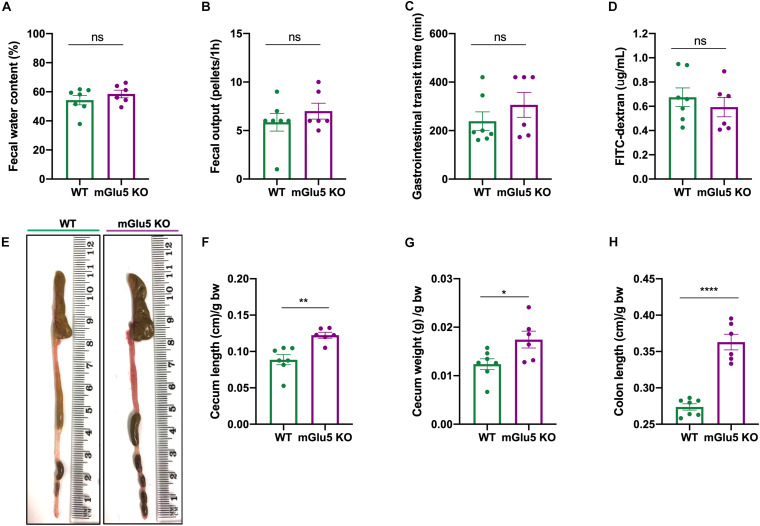
Gastrointestinal parameters of mGlu5 KO mice. **(A)** Fecal water content as a percentage of difference between the initial total feces weight and the dry weight. **(B)** Fecal output, counting the number of fecal pellets expelled during 1 h. **(C)** Gastrointestinal transit time, using the non- absorbable red dye Carmine red and recording first appearance in the feces. **(D)** Gut permeability measured by 4 kDa fluorescein isothiocyanate (FITC)-dextran assay. **(E)** Macroscopic evaluation of intestine. **(F)** Cecum length, **(G)** cecum weight, and **(H)** colon length normalized by body weight, unpaired *t*-test. The plots show mean ± SEM (*n* = 6–7 for both WT and mGlu5 KO mice groups). *p < 0.05, **p < 0.01, ***p < 0.001, and ns *p* > 0.05.

We did not observe any genotype difference in fecal water content (Figure 7A; *t* = 1.002, df = 11, *p* = 0.33), fecal output (Figure 7B; *t* = 0.91, df = 11, *p* = 0.37), gastrointestinal transit time (Figure 7C; *t* = 1.06, df = 11, *p* = 0.3) or gut permeability (Figure 7D; *t* = 0.72, df = 11, *p* = 0.48). On the other hand, we observed macroscopic differences between mGlu5 KO and WT mice (Figure 7E), including an increase in cecum length (Figure 7F; *t* = 4.02, df = 11, *p* = 0.002) and weight (Figure 7G); *t* = 2.52, df = 11, *p* = 0.02), together with an increase in colon length (Figure 7H; *t* = 8.12, df = 11, *p* < 0.0001), all relative to body weight. We did not find any macroscopic difference before normalizing to the body weight (Supplementary Figure 2).

## Discussion

This study provides the first evidence that an animal model of SZ replicates the gut dysbiosis observed in patients. In this study, we performed gut microbiome profiling of the mGlu5 KO and WT mice, by 16S rRNA sequencing of fecal samples. We found a significant genotype difference in beta diversity, indicating that mGlu5 KO and WT mice show differences in microbial communities. Specifically, using ANCOM, we uncovered differences in the relative abundance of *Erysipelotrichaceae* family and *Allobaculum* genus. We also identified a signature of bacteria discriminating both genotypes, consisting of the bacterial Order Erysipelotrichales, Bacteroidales, and Clostridiales. This new evidence for gut dysbiosis in mGlu5 KO mice provides unique opportunities to explore new approaches to improve treatment for SZ.

There are interesting overlaps between our findings in mice and clinical findings. Similar to our results, previous microbiome profiles of SZ patients have shown no difference between the most abundant phyla Firmicutes and Bacteroidetes (reviewed by Epskamp et al., 2012). Another parallel between our data and clinical SZ findings is that there is no difference in alpha diversity, but a significant difference in beta diversity between SZ patients and controls (Shen et al., 2018; Nguyen et al., 2019). However, it is important to note that while we found a significantly different beta diversity between mGlu5 KO and WT mice, the averaged distance-based coefficient of determination (*R*^2^ of the Bray Curtis and UniFrac results) was approximately 0.2, indicating that only around 20% of the microbiome variation is associated with the genotype. Still, a highly diverse gut microbiome is considered an advantage, being related to a healthy environment and lifestyle, usually associated with low or absent pathogenic bacterial species and also associated with improved cognition (e.g., learning, memory and behavioral flexibility; reviewed by Davidson et al., 2018). Therefore, our finding showing a subtle but significant differential beta diversity in mGlu5 KO mice relative to their WT littermate controls indicates a global differential community composition, and thus gut dysbiosis in this mouse model of SZ.

A highly diverse, as well as a balanced composition, of the microbial community is crucial to the homeostasis of the gut microbiome. Gut bacteria regulate basic processes such as digestion, maintenance of intestinal epithelium integrity and, importantly, commensal bacteria promote the first immune response against pathogenic bacteria (Rinninella et al., 2019). Furthermore, gut bacterial diversity and community composition determine the abundance of SCFAs, end-products of microbial fermentation which are vital for gut health (Campbell et al., 2016). One limitation of our study is that we had a relatively low sequencing depth at the alpha rarefaction curve, which could have influenced the alpha diversity analysis specifically. However, when we conducted an analysis investigating the microbial diversity for each sample at various sampling depths, we observed that in all cases the alpha diversity between the two genotypes was not significantly different (Supplementary Table 2).

When comparing humans and mice, there are extensive similarities in anatomy, physiology and genetics which have allowed numerous inferences about human health and pathologies. In gut microbiota research, mouse models have been increasingly used to study the role of the gut microbiota and its association with diseases (Nguyen et al., 2015). However, there are intrinsic differences between these two mammalian systems that make absolute comparisons challenging, particularly at the species level (Hugenholtz and de Vos, 2018). When care is taken in drawing direct parallels between murine and human gut, with regard to microbiota composition, mouse models provide valuable tools to study microbiota imbalance or dysfunction. Thus, our main goal with this study was to establish whether our mouse model of SZ provided an appropriate tool for preclinical studies of SZ. Our findings in the present study demonstrate that these mGlu5 KO mice may model the gut microbiota differential community composition observed in patients.

Using ANCOM we observed a decrease in the relative abundance of the family *Erysipelotrichaceae* in mGlu5 KO mice while the order Erysipelotrichales was one of the associated bacteria-signature discriminating mGlu5 KO and WT mice. In contrast, the same bacterial family was increased in adult mice born from a maternal immune activation (MIA) animal model of gestational influenza virus infection (Saunders et al., 2020) and a species from this family, *Catenibacterium mitsuokai*, was found relatively more abundant in SZ oral samples than in controls (Castro-Nallar et al., 2015). ANCOM analysis also indicated a decrease in the genus *Allobaculum* in mGlu5 KO mice when compared to WT. This genus is a member of the family *Erysipelotrichaceae* and is associated with a healthy microbiome, suggested to have beneficial gut effects related to mucus properties and formation (Everard et al., 2014). A decrease in the abundance of *Allobaculum* has been observed in the APP/PS1 mouse model of Alzheimer’s disease (Harach et al., 2017) and absent in restrained mice as a depression-like animal model, while high in the control group (Koo et al., 2010). Importantly, the treatment with the antipsychotic risperidone in mice was shown to increase the abundance of *Allobaculum* (Bahr et al., 2015).

The order Bacteriodales was one of our signature bacteria responsible for discriminating our genotypes (mGlu5 KO versus WT mice) and it was previously associated with individuals with ultra-high risk for psychosis, when compared to healthy controls (He et al., 2018). In addition, the genus of the same taxa *Bacteroides*, was associated with SZ in a recent gene set enrichment analysis (GSEA) study that used published psychiatric disorder GWAS data, as well as GWAS of gut microbiota summary data (Cheng et al., 2020). Clostridiales by its turn, another signature bacteria responsible to discriminate our SZ mouse model from control WT mice in our study, was also already associated with ultra-high risk for psychosis individuals when compared to healthy controls (He et al., 2018). Families from this order were demonstrated to be decreased in SZ patients, suggesting an impairment in the maintenance of gut health (Zheng et al., 2019). Other studies also demonstrated an association between lower levels of the Clostridiales order in SZ patients (Shen et al., 2018). Furthermore, a negative correlation between *Clostridium_sensu_stricto_1* and the positive and negative syndrome scale (PANSS) score, which is a medical scale used for measuring symptom severity of patients with SZ, was recently reported, suggesting that changes in intestinal microbiota may modulate the prognosis of the disorder (Pan et al., 2020). We also performed a co-occurrence network analysis using SparCC to identify associations between the microbes. We observed that the main hubs (or keystone taxa) of the network are not microbes detected by sPLS-DA. The microbes identified by sPLS-DA are clustered together and their interactions are mainly local (within their own neighborhood). Some of these sPLS-DA identified microbes are also articulation points, in which they bridge the connection between two separate neighborhoods. However, we acknowledge that this approach may be insufficient in inferring microbial community structure (Hirano and Takemoto, 2019).

We have therefore identified interesting overlaps between our present findings and previous clinical SZ literature, with promising translational bacterial targets. However, it is important to note that, regarding specific taxa abundance and enrichment, the discrepancies between clinical studies are even more prominent, mainly due to methodological differences and inherent limitation of the clinical heterogeneity of SZ (Macedo e Cordeiro et al., 2020). Therefore, a simple gut microbiome signature for SZ patients is still not determined. It is essential in clinical studies to rigorously control for treatment, diet, physical activity and previous prebiotic/probiotic/antibiotic use, among other confounders; however, this is not always possible. Nevertheless, the fully controlled environment (as well as genetic control) that animal studies provide can overcome these limitations, and preclinical studies that replicate a general dysbiosis observed in patients, including this mGlu5 KO mouse model, represent a useful tool for the study of the gut microbiome in psychiatric disorders.

A few studies with animal models of relevance to SZ have previously suggested a promising potential of a microbial community role associated to these preclinical models. Sub-chronic N-methyl-D-aspartate receptor (NMDAR) antagonist phencyclidine (PCP) administration in male Lister Hooded rats resulted in cognitive impairment and differential beta diversity (Pyndt Jørgensen et al., 2015). A correlation between gut microbiota and memory performance was also demonstrated, in addition to the administration of antibiotic being able to reverse the cognitive deficits observed in sub-chronic PCP treated rats (Pyndt Jørgensen et al., 2015). However, this last result should be carefully interpreted since they did not investigate the direct central effect that ampicillin is able to perform, and thus this antibiotic may have acted directly on the brain rather than via the gut (Nau et al., 2010). Similarly, in an MIA model of maternal influenza viral infection and associated neurodevelopmental disorder, cognitive impairments were observed in the offspring and antibiotic administration during their prepuberal period was able to prevent that outcome (Saunders et al., 2020). Additionally, the same study showed that adult MIA offspring displayed altered gut microbiota, with differential relative abundance between components of the gut microbiota, including *Ruminococcaceae*. Likewise, in Wistar rats, an animal model combining MIA with polyI:C and adolescent cannabinoid exposure demonstrated a sex-specific effect, with adolescent female offspring exhibiting decreased fecal levels of *Bifidobacterium longum*, measured using qPCR (Katz-Barber et al., 2020). While very promising, these aforementioned studies should be interpreted with caution, since recapitulating the complexity of SZ in an animal model is still a challenge, and each study has its own limitations. In particular, the PCP model lacks construct validity (Sams-Dodd, 1999) and MIA animal models are associated with a broad range of disorders, including ASD, and thus MIA may be considered to be a non-specific primer of multiple neurodevelopmental disorders (Estes and McAllister, 2016).

The genetics of SZ is complex and heterogeneous and thus genome-wide association studies (GWAS) do not identify single mutations strongly associated with this psychiatric disorder. However, mGlu5 has been implicated in SZ pathogenesis (Devon et al., 2001; Gupta et al., 2005), along with many related proteins associated with synapse development and function. Decreased mGlu5 mRNA and protein levels in SZ patients have been reported in postmortem studies (Volk et al., 2010; Matosin et al., 2015). The mGlu5 KO mice demonstrate impaired prepulse inhibition (PPI) (Brody and Geyer, 2004; Burrows et al., 2015), baseline hyperactivity and a hypersensitivity to MK-801-induced hyperlocomotion (Lipina et al., 2007; Gray et al., 2009), impaired circadian process (Aguilar et al., 2020), clinically relevant cognitive deficits on touchscreen tasks (Zeleznikow-Johnston et al., 2018) and thus excellent face validity as a preclinical model of SZ. Lastly, chronic treatment with clozapine is able to reverse SZ-related behaviors (Gray et al., 2009), demonstrating strong predictive validity. Taken together, these studies indicate that mGlu5 KO mouse model has great utility for the study of SZ, including the altered microbiome associated with gut dysbiosis.

Recently, a study focused on the association between depressive behavior and the mGlu5 receptor reported 16S rRNA sequencing in feces from another line of mGlu5 KO mice, concluding that there were no gut microbiota changes between genotypes (Cai et al., 2020). Discrepancies between this study and ours are likely to result from multiple factors, including differences in the generation and genetic background of mGlu5 KO mice. In their study, Cai and colleagues generated mGlu5 KO mice by crossing Grm5^*flox/flox*^ mice with B6.C-Tg (CMV-cre) mice but did not explore SZ-like endophenotypes. Our Grm5tm1Rod line of mice have a constitutive null mutation in the mGlu5 gene, with strong face and predictive validity as a preclinical model of SZ (Hannan et al., 2001; Gray et al., 2009; Burrows et al., 2015). In addition, Cai and collaborators have not extensively analyzed their microbiome data. In particular, similar to their study, we didn’t observe any difference between alpha diversity, but when we analyzed other levels of complexity, such as beta diversity and sPLS-DA (low classification error rate), we discovered a genotype effect. Therefore, following thorough and exhaustive microbiome profiling, our present findings are the first to demonstrate gut dysbiosis in the mGlu5 KO mouse model of SZ.

Consistent with previous findings (Bradbury et al., 2005), we also observed a reduced body weight in mGlu5 KO mice when compared to WT controls, despite no changes in food and water intake between groups. In fact, mGlu5 has been suggested to be a mediator of energy balance in rodents by decreasing caloric efficiency, suggesting increased energy expenditure in mGlu5 KO mice (Bradbury et al., 2005). Corroborating these findings, it was also demonstrated that activation of lateral hypothalamic mGlu5 receptors elicits feeding in rats (Ploj et al., 2010; Charles et al., 2014) and mGlu5 KO mice are resistant to diet-induced obesity (Bradbury et al., 2005). Interestingly, the abundance of the genus *Allobaculum*, which we have found decreased in mGlu5 KO mice, has been correlated with body weight and dietary-induced inflammation markers, including leptin and IL-22 (Ravussin et al., 2012; Zenewicz et al., 2013; Everard et al., 2014). Considering the highly explored and relevant role of gut microbiota composition in the pathogenesis of obesity (Leocádio et al., 2020), our novel findings showing a differential gut microbiome profile between mGlu5 KO and WT mice may have relevance to the study of metabolic and eating disorders, including obesity.

Glutamate is a central and peripheric modulator, with glutamatergic dysfunction associated with both central nervous system (CNS) disorders and GI diseases, which in many cases display intercorrelated co-morbidities (e. g. inflammatory bowel disease (IBD) and depression) (Keefer and Kane, 2017; Baj et al., 2019). Not surprisingly, SZ is also associated with GI issues, such as gut inflammation and gut cellular barrier defects (Severance et al., 2015). In fact, the glutamatergic system is directly implicated in GI modulation, with the mGlu5 receptor being also expressed in the GI tract (Ferrigno et al., 2017) and involved with the peripheral excitatory modulation of vagal afferent mechanosensitivity (Slattery et al., 2006). Interestingly, antipsychotics, the main treatment for SZ, induce weight gain, constipation and metabolic syndrome (Kanji et al., 2018; Zeng et al., 2020). It is possible that the negative side effects of antipsychotics may be at least partly related to an antibiotic-like side effect on gut microbiota, by altering its composition and decreasing diversity (reviewed in (Dinan and Cryan, 2018; Skonieczna-Źydecka et al., 2019)). Thus, microbiota interventions adjunctively with antipsychotics are promising to alleviate adverse side effects and mGlu5 KO mice may provide a useful preclinical tool to investigate these and other aspects of SZ pathogenesis and treatment.

Considering that the interactions between microbiota composition and gut status are bidirectional, with the bacteria population playing an important role in gut function and the gut function affecting the diversity of the microbiome in the GI tract (Barbara et al., 2005), we evaluated GI health parameters. We didn’t see any difference in fecal water content, fecal output or gastrointestinal transit time, which have been interrelated with gut microbiota composition (Kashyap et al., 2013; Vandeputte et al., 2016). Similarly, we didn’t see a difference in gut permeability, which has been linked to dysbiosis and increased inflammation (Chakaroun et al., 2020). One possible explanation is that, due to the fact that only around 20% of the microbiome variation revealed by beta diversity is associated to the genotype, dysbiosis can potentially not be strong enough to modulate or be reflected in the gastrointestinal parameters analyzed. On the other hand, after normalization by body weight we observed macroscopic differences between mGlu5 KO and WT mice, including an increase in cecum length and weight, together with an increased colon length. Since colon length can be considered a marker of colonic inflammation (Chassaing et al., 2014) and cecum measurements also associates with gut microbiota composition (Ge et al., 2017) further study is needed to explore whether mGlu5 KO mice present gastrointestinal dysfunction associated with inflammation. Therefore, clarifying the extent of the gut dysbiosis in this mGlu5 KO mouse model and establishing whether gut dysbiosis is causally associated with GI dysfunction, would be of great interest.

## Conclusion

In conclusion, the microbiome phenotype observed in mGlu5 KO mice is to some extent in line with reports of gut dysbiosis in SZ patients, and thus this animal model provides a novel tool to explore the mechanistic understanding of how and when dysbiosis arises in SZ. Furthermore, our present findings can be used to explore the link between dysbiosis and SZ symptoms (i.e., behavioral impairments) as well as the potential utility of gut microbiome restoration as a therapeutic approach (e.g., fecal matter transplantation, probiotics and prebiotics), while clarifying the extent of microbiota–gut–brain axis dysfunction in this model. Moreover, considering that mGlu5 KO mice also present deficits in basic discrimination learning and cognitive flexibility (Zeleznikow-Johnston et al., 2018), this mouse model can be also used as a tool for the investigation of the role of the gut microbiome in generalized cognitive impairments. Thus, our present findings may also inform other cognitive disorders where mGlu5 receptors play a role and the microbiome association is still not well understood, such as addiction (Terbeck et al., 2015; Meckel and Kiraly, 2019).

Our results provide the first evidence that a genetic animal model of SZ, exhibiting both face and predictive validity, at least partly replicates the gut dysbiosis observed in patients. These new findings of gut dysbiosis in the mGlu5 KO mice provide opportunities to explore novel approaches focusing on the microbiota–gut–brain axis in SZ. We therefore propose that this preclinical model of SZ can be used as a tool to investigate how gut dysbiosis may contribute to SZ pathogenesis, via the microbiota–gut–brain axis. Our new findings may also inform the development of novel therapeutic approaches for SZ, a devastating disorder for which new treatments are urgently needed.

## Data Availability Statement

The datasets and metadata related to this study have been deposited in the NCBI Sequence Read Archive under BioProject number PRJNA659149. Furthermore, the R code for the analysis has been uploaded to the following site: https://github.com/gkong1/mGLU5KO_microbiome_analysis.

## Ethics Statement

The animal study was reviewed and approved by the Florey Institute of Neuroscience and Mental Health Animal Ethics Committee.

## Author Contributions

CG was involved in the experimental design, data collection, data analysis, and manuscript writing. GK was involved in microbiome data analysis. VU, AZ-J, EB, and TR were involved in the data collection, analysis, and editing of the manuscript. AH was involved in the experimental design, project management and funding, data analysis, and drafting of the manuscript. All authors contributed to the article and approved the submitted version.

## Conflict of Interest

The authors declare that the research was conducted in the absence of any commercial or financial relationships that could be construed as a potential conflict of interest.
