# Supplementary material for: Microbiome Profiling Reveals Gut Dysbiosis in the Metabotropic Glutamate Receptor 5 Knockout Mouse Model of Schizophrenia
Source: Front Cell Dev Biol. 2020 Oct 29;8:582320. doi: 10.3389/fcell.2020.582320 (PMC7658610; doi:10.3389/fcell.2020.582320)
Supplement: Supplementary file 1 [file Image_1.pdf]

Supplementary Figure 1

Microbiome profiling reveals gut dysbiosis in the metabotropic glutamate receptor 5 knockout mouse model of schizophrenia

Gubert et al.

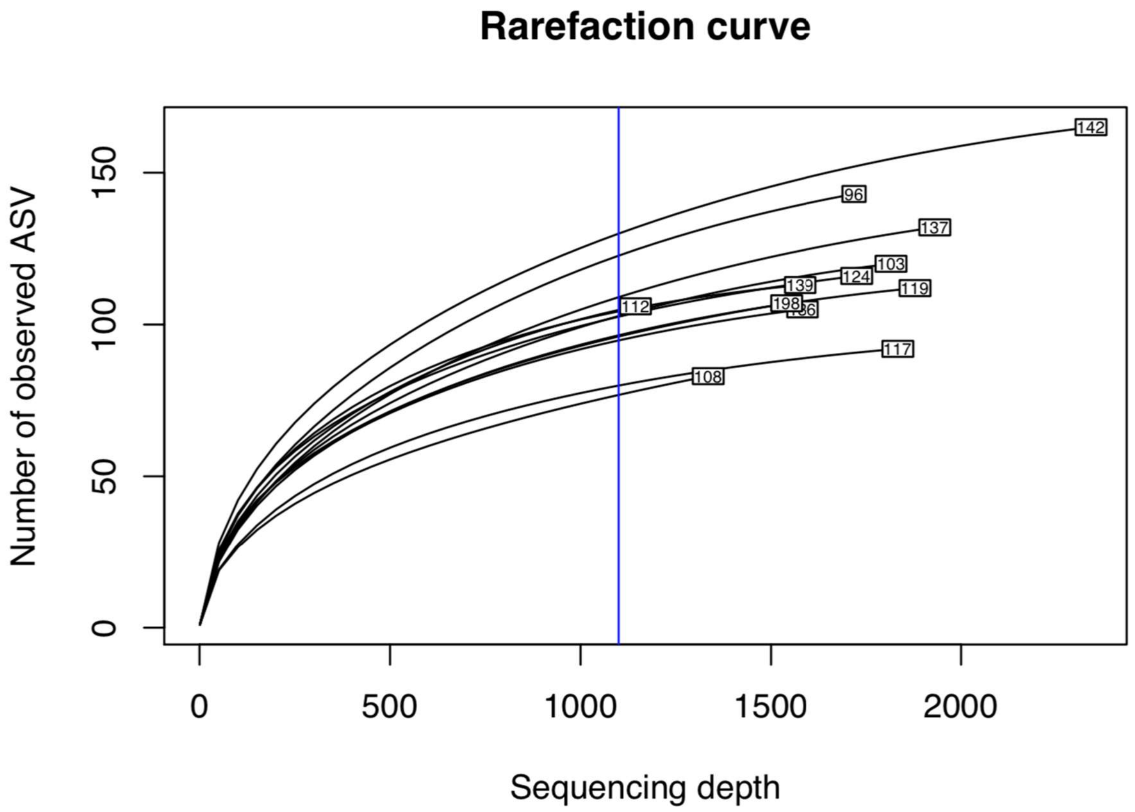

Suppl. Fig. 1. Alpha rarefaction plot of all samples rarefied to 1,100 reads per sample. It Represents species richness as a function of the number of reads (n = 6 for both WT and mGlu5 KO mice groups).
