# Supplementary material for: Microbiome Profiling Reveals Gut Dysbiosis in the Metabotropic Glutamate Receptor 5 Knockout Mouse Model of Schizophrenia
Source: Front Cell Dev Biol. 2020 Oct 29;8:582320. doi: 10.3389/fcell.2020.582320 (PMC7658610; doi:10.3389/fcell.2020.582320)
Supplement: Supplementary file 2 [file Image_2.pdf]

Supplementary Figure 2

Microbiome profiling reveals gut dysbiosis in the metabotropic glutamate receptor 5 knockout mouse model of schizophrenia

Gubert et al.

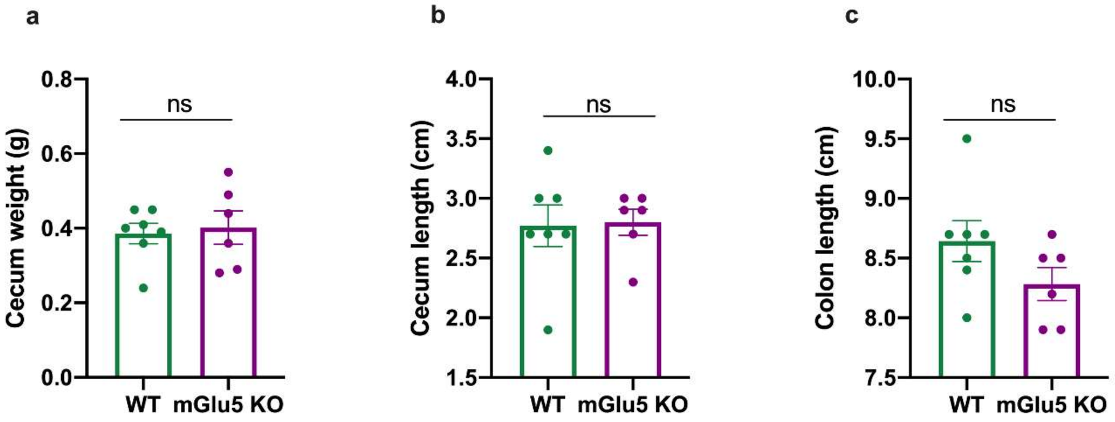

Suppl. Fig. 2. Gastrointestinal parameters of mGlu5 KO mice. Macroscopic evaluation of intestine. **(a)** Cecum weight, **(b)** cecum length and **(c)** colon length without normalization by body weight; unpaired t-test. The plots show mean ± SEM (n = 6-7 for both WT and mGlu5 KO mice groups). ns $p>0.05$.
