# Supplementary material for: Microbiome Profiling Reveals Gut Dysbiosis in the Metabotropic Glutamate Receptor 5 Knockout Mouse Model of Schizophrenia
Source: Front Cell Dev Biol. 2020 Oct 29;8:582320. doi: 10.3389/fcell.2020.582320 (PMC7658610; doi:10.3389/fcell.2020.582320)
Supplement: Supplementary file 3 [file Table_1.pdf]

Supplementary Table 1

Microbiome profiling reveals gut dysbiosis in the metabotropic glutamate receptor 5 knockout mouse model of schizophrenia

Gubert et al.

Suppl. Table 1. Quality control metrics. Number of sequences (n = 6 for both WT and mGlu5 KO mice groups).

DMG Number	Submitter ID	Number of Sequences
DMG1904020	mGlu5.WT.103	77591
DMG1904021	mGlu5.WT.119	80769
DMG1904022	mGlu5.WT.112	58880
DMG1904023	mGlu5.WT.96	77132
DMG1904024	mGlu5.WT.137	84643
DMG1904025	mGlu5.WT.142	105554
DMG1904026	mGlu5.KO.136	68913
DMG1904027	mGlu5.KO.139	67896
DMG1904028	mGlu5.KO.124	78487
DMG1904029	mGlu5.KO.198	75584
DMG1904030	mGlu5.KO.108	63400
DMG1904031	mGlu5.KO.117	73036
